# Identification and testing of sex pheromone components of the invasive Australian redback spider (*Latrodectus hasseltii*)

**DOI:** 10.1038/s41598-026-49837-w

**Published:** 2026-04-27

**Authors:** Andrew M. Twidle, Thomas E. S. Sullivan, Meikura T. Arahanga, Lisa I. Pilkington, Devon T. Bryant, Nigel I. Joyce, Nicola J. Sullivan, Tara J. Murray, Cor J. Vink

**Affiliations:** 1 New Zealand Institute for Bioeconomy Science Limited, Christchurch Mail Centre, Private Bag 4704, Christchurch, 8140 New Zealand; 2 Bioprotection Aotearoa, Lincoln University, PO Box 85084, Lincoln, 7647 New Zealand; 3 School of Chemical Sciences, University of Auckland, Private Bag 92019, Auckland, New Zealand; 4 Te Pūnaha Matatini, Auckland, 1142 New Zealand; 5 Department of Pest-Management and Conservation, Lincoln University, PO Box 85084, Lincoln, 7647 New Zealand; 6 Department of Conservation (DOC), PO Box 5244, Dunedin, 9054 New Zealand

**Keywords:** Spider pheromone, Pheromone trapping, Pest management, Pest surveillance, Carboxylic acids, Serine derivatives, Biochemistry, Chemical biology, Chemistry, Ecology, Ecology, Zoology

## Abstract

Australian redback spiders *Latrodectus hasseltii*, known for their strong neurotoxic venom, are a quarantine threat for much of the world. Female redback spiders produce a sex pheromone that attracts males of the species, yet the compounds involved in their attraction remain unresolved. Our project set out to identify these compounds and use them to trap male redback spiders as the first step in the development of a pest management/surveillance tool for these spiders. Headspace volatiles from the silk of virgin and mated female redback spiders was collected by solid phase microextraction and analysed by gas chromatography coupled with mass spectrometry. Silk samples were also solvent extracted for analysis by liquid chromatography coupled with mass spectrometry. Comparison with synthetic standards on multiple column phases identified the candidate volatile pheromone components as 2-methylpropanoic acid, (*S*)-2-methylbutanoic acid and 2-pyrrolidone, while *N*-3-methylbutanoyl-*O*-(*S*)-2-methylbutanoyl-L-serine was identified as the main candidate short-range cue. Bioassays with a combination of these four compounds showed similar levels of attraction to the natural virgin female’s silk. Field trapping trials using a mixture of all four compounds successfully trapped male redback spiders in New Zealand vineyards.

**Supplementary Information:**

The online version contains supplementary material available at 10.1038/s41598-026-49837-w.

## Introduction

Australian redback spiders, *Latrodectus hasseltii* Thorell, 1970 (Araneae: Theridiidae) are invasive pests in Japan and New Zealand^1^. They have also been reported in Papua New Guinea^2^(Vink unpublished data) and central Europe^3^. The ability of redback spiders to tolerate a temperature range from below 0 °C^4^ to 40 °C^5^, survive without food for months^6^, successfully associate with human dwellings^5^, and mate with their siblings without inbreeding depression^7^, means they represent a quarantine threat to much of the world^1^. Known for their strong neurotoxic venom, redbacks pose a risk to humans and native fauna^5,8^ in any invaded environment. Unlike most spider species, redbacks produce a toxin that specifically targets vertebrates, α-latrotoxin^9^, with their prey including lizards and small mice^5^. α-Latrotoxin is a potent neuro-disruptor^10^, whose effects can be life-threatening in humans, which has led to hospitals in their invaded ranges stocking antivenom^10^. Since the advent of redback antivenom in 1956 there has only been one death associated with a redback spider bite in 2016 and this was suspected to be from a secondary infection rather than the venom itself^11^. Outside of traditional pesticide sprays there are no specific control measures for these pest spiders and there are no means to specifically test for their presence.

Spider pheromones have received little attention in the scientific literature with only 21 species having sex pheromone candidate compounds identified to date^12^ from the more than 53,700 known spider species^13^. Insects on the other hand have had thousands of communication compounds identified^12^, and their use is widespread in pest management^14^ and surveillance^15^. Spiders are mostly beneficial arthropods predating on pest insect species and providing positive ecosystem services^16^, which is a likely explanation for this difference in the level of study. However, with globalisation, the rate of unintentional introductions of pest spiders is increasing^17^, which in turn raises the risk of a pest population becoming established. For the redback spiders this is particularly poignant due to their characteristics described above, and the fact that a single gravid female can produce > 1200 offspring (unpublished data), meaning that one mated female invading a new environment is capable of establishing a viable population.

Behavioural studies have shown that virgin female redback spiders use sex pheromones deposited on the silk of their web for mate attraction and that just-mated females do not^18,19^. Initial work on compound identification using methanol extracts of virgin silk reported that redback spiders produce a contact pheromone *N*-3-methylbutanoyl-*O*-(*S*)-2-methylbutanoyl-L-serine methyl ester^20^. More recently butanoic acid and 3-methylbutanoic acid were reported as putative airborne pheromone components for redback spiders from laboratory trials^21^, but were unable to display any field attraction (pers. comm.). In the wider Theridiidae family a range of related short chain acids and serine derivatives have been reported as pheromone components^22–24^. For the false widow *Steatoda grossa* (Araneae: Theridiidae) it has been shown that the contact pheromone components are broken down to the airborne components via a carboxyl ester hydrolase enzyme^22^. However there have been very limited reports of successful field captures using these synthetic pheromones, with a range of carboxylic acid components only capturing a total of nine male false widow spiders over four months of trapping^22^, while in another trial focusing on *L. hesperus*, a single component lure of isobutyric acid caught a total of six male spiders^24^.

Very few spider pheromones have been identified worldwide and their use as pest management tools has not been reported. With the previously identified redback compounds inactive in the field (pers. comm.), our aim for this project was to re-investigate the female produced sex pheromone of the redback spider to identify the attractive components for use as a potential trapping tool for this invasive pest. This coupled with redback male self-sacrifice during mating^18,25^ presents the opportunity to control redback populations by reducing the number of males. Here we report the analysis of the redback spider female’s silk, identification of candidate compounds, then their successful testing in the laboratory and field environments as the first step in the development of a pest management and surveillance tool.

## Methods

### Spiders

Redback spiders and egg sacs were collected from the Alexandra, Cromwell and Bannockburn regions in the South Island of New Zealand from December 2023 to February 2024. Spiders and egg sacs were individually housed upon their capture, as were spiderlings once they emerged from the egg sacs. All spiderlings up to 4th moult were housed in small cylindrical chambers (30 mm D x 70 mm L) until they could be sexed. The larger females evident from the 5th moult onwards were housed in cuboid chambers (160 mm x 110 mm x 110 mm). The much smaller male spiders were housed in the small cylindrical chambers only. The small cylindrical chambers contained a wooden ‘V’ for web construction while the larger cuboid containers held a wooden ‘cross’ for the spiders to construct their webs on. All spiderlings upto the 4th moult and all male spiders were fed 1–3 vinegar flies (*Drosophila melanogaster*) per week, while the larger females (5th moult onwards) were fed monthly with a mixture of live mealworms (*Tenebrio molitor*, late instar/adults) and crickets (*Teleogryllus commodus*). The laboratory containing the spiders was kept at 25°C under a 14 hr : 10 hr light-dark cycle, while humidity in the laboratory ranged from approx. 45–50%. Spiders were not mated in the laboratory. For the purposes of our trials mated females were collected directly from the wild. A mated female was determined as an adult female spider sitting on eggs when captured. Virgin females were reared through from spiderlings and were deemed as sexually mature once the epigynum was visible (when viewed under a microscope) and were used immediately for silk collection.

### Silk volatile analysis

Following feeding, the desiccated prey carcasses and old silk were removed and the mature adult female spiders were given a maximum of four weeks to construct a large web in their enclosure. Silk was collected from virgin adult females (*n* = 21) and mated adult females (*n* = 32) for headspace volatile analysis using solvent cleaned, oven baked (150 °C for 24 h) forceps and vials. Due to the large variation in silk production/web size between individual spiders, samples for headspace volatile sampling consisted of enough silk to approximately half fill the 4 mL amber glass collection vials (ranging from two to ten webs). Headspace volatiles from the silk were collected using a 75 μm CAR/PDMS solid phase microextraction (SPME) fibre (Supelco, Bellefonte, PA, USA) inserted into the vial and held directly above the silk. SPME fibres were conditioned for 30 min at 270 °C in an Agilent 7890B (Santa Clara, CA, USA) gas chromatograph (GC) injection port prior to use. SPME samples were collected for 1 h at 25 °C, then thermally desorbed for 10 min in the injection port of a GC coupled to an Agilent 5977 A mass spectrometer (MS). Helium was used as a carrier gas with a flow rate of 1.3 mL/min and injections were splitless. Samples were analysed on non-polar DB-5ms UI (30 m x 0.25 mm i.d. x 0.25 μm), polar DB-wax (30 m x 0.25 mm i.d. x 0.50 μm) and chiral Cyclosil B (30 m x 0.25 mm i.d. x 0.25 μm) columns, where each column was individually installed into the GC oven when required. The injector was set at 250 °C (220 °C for the polar and chiral columns) and the oven temperature programme started at 40 °C (2 min hold), then was increased at a rate of 4 °C/min up to 280 °C (230 °C and 220 °C for polar and chiral columns respectively). An electron impact ionization of 70 eV was used for the MS analysis.

### Silk extract analysis

Initial solvent extracts of the virgin female silk were conducted using methanol in line with the methods of those previously reported^20,22^. Here the silk from 10 webs was covered with methanol (ca. 2.5 mL) for 24 h after which time the supernatant was decanted off and concentrated for analysis by GC-MS. The silk extracts were compared with synthetic standards on the three column types to confirm their identity. A sample of the methanol silk extract was also methylated using acidified methanol to visualise any carboxylic acids that were not eluting under the GC-MS conditions described above. This consisted of taking the methanol silk extract above (ca. 250 µL), adding 1 µL of H_2_SO_4_ then leaving the solution to react for 90 min at room temperature. After this time the reaction was quenched with Na_2_CO_3_ (aq) and the methylation products were extracted with 2 × 250 µL of dichloromethane (DCM). The resulting solution was dried over MgSO_4_ and concentrated under Ar for GC-MS analysis. Following on from the methylation, a new extract was made from fresh virgin silk (10 webs) using acetonitrile as the solvent, which was then analysed by liquid chromatography (LC) coupled to MS to help visualise the more polar compounds. Samples were analysed via reverse phase chromatography (Hypersil GOLD™ aQ C18 1.9 μm, 100 mm x 2.1 mm, Thermo Scientific) using a Thermo Scientific™ (San Jose, CA, USA) Vanquish™ UHPLC system (Binary Pump H, Split Sampler HT, Dual Oven) coupled with a Thermo Scientific™ (San Jose, CA, USA) Q Exactive™ Plus Orbitrap (version Orbitrap MS 2.11 build 3007). The mobile phase consisted of 0.1% formic acid in type 1 water (A) and 0.1% formic acid in acetonitrile (B) maintained at 30 °C with a flow rate of 300 µL/min. A gradient was applied: 0–1 min/0% B, linear increase to 2 min/20% B, linear increase to 8 min/40% B, linear increase to 10 min/98% B, isocratic to 13 min/98% B, linear decline to 14 min/0% B, isocratic to end 17 min/0% B. The eluent was scanned from 0.5 to 13.5 min by API-MS (Orbitrap) with heated electrospray ionization at 150 °C and capillary temperature 350 °C in the negative and positive mode. Data was acquired for precursor masses *m*/*z* 80–600 amu at 140 K resolution (AGC target 1e6, maximum IT 100 ms, profile mode) with data dependent ms/ms for product ions generated by normalised collision energy NCE:25, 45, 85 and at 17.5 K resolution (TopN 10, AGC target 1e5, Maximum IT 50 ms, Isolation 1.4 *m*/*z*). Compounds were confirmed by comparison with synthetic standards.

### Chemicals

**Fig. 1:**
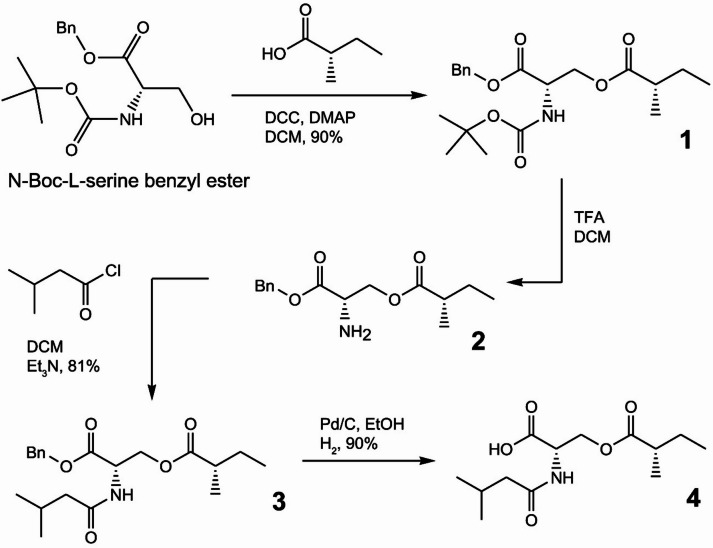
Synthesis of *N*-3-methylbutanoyl-*O*-(*S*)-2-methylbutanoyl-L-serine (**4**), potential short-range attractant of the Australian redback spider *Latrodectus* *hasseltii*.

Solvents and commercially available compounds used for the silk extractions, bioassays and field testing were supplied by Sigma-Aldrich (St. Louis, MO, USA) in the purity listed (%): methanol HPLC grade (> 99.9%), DCM LiChrosolv^®^ grade (> 99.9%), acetonitrile LiChrosolv^®^ grade (> 99.9%), 2-methylpropanoic acid (99%), 2-methylbutanoic acid (98%), (*S*)-2-methylbutanoic acid (98%) and 2-pyrrolidone (99%). The *N*-3-methylbutanoyl-*O*-(*S*)-2-methylbutanoyl-L-serine was synthesised in-house (Fig. 1) and is described in the text below. The synthesis of 3-methylbutanoyl chloride, *N*-3-methylbutanoyl-*O*- 2-methylpropanoyl-L-serine and *N*-3-methylbutanoyl-*O*-(*S*)-2-methylbutanoyl-L-serine methyl ester are outlined in the supplementary information. All of the reagents used for synthesis were obtained from Sigma-Aldrich (St. Louis, MO, USA) and were used as supplied without further purification unless otherwise stated. The identity of the synthesised compounds were initially checked by MS and confirmed by nuclear magnetic resonance (NMR) on a 400 MHz spectrometer. Chemical shifts are reported relative to the solvent peak of chloroform and/or CDCl3 (δ 7.3 for ^1^H and δ 77.0 for ^13^C, respectively). ^1^H NMR data is reported as position (δ), relative integral, multiplicity (s, singlet; d, doublet; t, triplet; dd, doublet of doublets; dt, doublet of triplets; m, multiplet; br, broad peak; sext, sextet; q, quartet; sept, septet), coupling constant (*J*, Hz), and the assignment of the atom. ^13^C NMR data are reported as position (δ) and assignment of the atom. NMR assignments were performed using HSQC and HMBC experiments. High resolution MS (HRMS) was carried out by electrospray ionisation (ESI) on an Orbitrap Exploris 120 mass spectrometer.

### Pheromone synthesis

#### *N*-Boc-*O*-(*S*)-2-methylbutanoyl-L-serine benzyl ester (**1**)

To a stirred solution of *N*-Boc-L-serine benzyl ester (1.50 g, 5.08 mmol) in dry DCM (50 mL) at 0 °C, (*S*)-2-methylbutanoic acid (0.57 g, 5.58 mmol) was added. This was followed by the addition of 4-dimethylaminopyridine (0.134 g, 1.10 mmol) and dicyclohexylcarbodiimide (1.17 g, 5.67 mmol). The resulting mixture was allowed to warm to room temperature, then stirred for 48 h. Upon completion the dicyclohexylurea precipitate was filtered off and the filtrate was washed with a saturated solution of NaHCO_3_ (20 mL) then dried (MgSO_4_). The solvent was removed *in vacuo* and the crude product was purified by column chromatography (silica gel, hexane: ethyl acetate gradient) to give ester (**1**) (1.74 g, 4.59 mmol, 90% yield). ^1^H NMR (400 MHz; CDCl_3_; Me_4_Si) δ_H_: 0.86 (3 H, t, *J* = 7.5 Hz, CH_2_C*H*_3_), 1.05 (3 H, d, *J* = 7.0 Hz, CHC*H*_3_), 1.44 (9 H, s, C(C*H*_3_)_3_), 1.54–1.66 (2 H, m, C*H*_2_CH_3_), 2.29 (1H, sext., *J* = 7.0 Hz, C*H*CH_3_), 4.31 (1H, dd, *J* = 3.4 and 11.0 Hz, OC*H*_2a_CH), 4.47 (1H, dd, *J* = 4.0 and 11.0 Hz, OC*H*_2b_CH), 4.58–4.64 (1H, m, NHC*H*), 5.17 (2 H, d, *J* = 8.0 Hz, C*H*_2_-Ar), 5.28 (1H, br d, *J =* 8.2 Hz, NH), 7.38–7.30 (5 H, m, Ar*H*). ^13^C NMR (100 MHz; CDCl_3_) δ_C_: 11.6 (CH_2_*C*H_3_), 16.6 (CH*C*H_3_), 26.6 (*C*H_2_CH_3_), 28.5 (C(*C*H_3_)_3_), 40.3 (*C*HCH_3_), 53.2 (NH*C*H), 64.2 (O*C*H_2_CH), 67.5 (Ar-*C*H_2_), 80.3 (*C*(CH_3_)_3_), 128.3 (*C*H-Ar), 128.5 (*C*H-Ar), 128.6 (*C*H-Ar), 135.0 (*C*-Ar), 169.8 (NHCH*C*OO), 174.9 (NH*C*OO), 176.2 (CH_3_CH_2_CH(CH_3_)*C*OO). HRMS (ESI^+^): Found (MH^+^): 380.2067, C_20_H_29_NO_6_ requires 380.2068.

#### *N*-Hydro-*O*-(*S*)-2-methylbutanoyl-L-serine benzyl ester (**2**)

To a stirred solution of ester (**1**) (1.71 g, 4.51 mmol) in dry DCM (25 mL), trifluoroacetic acid (5 mL) was added dropwise at room temperature. After 2 h of mixing, the solvent, remaining acid and other volatiles were removed *in vacuo*. The crude amino ester (2) was then characterized by NMR and used directly in the next step without purification. ^1^H NMR (400 MHz; CDCl_3_; Me_4_Si) δ_H_: 0.83 (3 H, t, *J* = 7.4 Hz, CH_2_C*H*_3_), 1.02 (3 H, d, *J* = 7.0 Hz, CHC*H*_3_), 1.33–1.44 (1H, m, C*H*_2a_CH_3_), 1.53–1.64 (1H, m, C*H*_2b_CH_3_), 2.32 (1H, sext., *J* = 7.0 Hz, C*H*CH_3_), 4.41–4.43 (1H, m, NH_2_C*H*), 4.54 (1H, dd, *J* = 2.4 and 12.8 Hz, OC*H*_2a_CH), 4.62 (1H, dd, *J* = 4.4 and 12.8 Hz, OC*H*_2b_CH), 5.22 (2 H, d, *J* = 9.4 Hz, C*H*_2_-Ar), 6.92 (2 H, br s, NH_2_) 7.31–7.38 (5 H, m, Ar*H*). ^13^C NMR (400 MHz; CDCl_3_; Me_4_Si) δ_C_: 11.2 (CH_2_*C*H_3_), 16.0 (CH*C*H_3_), 26.2 (*C*H_2_CH_3_), 40.5 (*C*HCH_3_), 53.1 (NH_2_*C*H), 61.3 (O*C*H_2_CH), 69.3 (Ar-*C*H_2_), 128.7 (*C*H-Ar), 128.8 (*C*H-Ar), 129.1 (*C*H-Ar), 133.6 (C-Ar), 166.4 (CHNH_2_*C*OO), 176.6 (CH_3_CH_2_CH(CH_3_)*C*OO). HRMS (ESI^+^): Found (MH^+^): 280.1543, C_15_H_21_NO_4_ requires 280.1543.

#### *N*-3-Methylbutanoyl-*O*-(*S*)-2-methylbutanoyl-L-serine benzyl ester (**3**)

Crude amino ester (**2**) (1.23 g, 4.40 mmol) was dissolved in dry DCM (25 mL) and triethylamine (2.05 g, 20.26 mmol) was added dropwise at 0 °C under constant stirring. 3-Methylbutanoyl chloride (1.36 g, 11.28 mmol) was then added dropwise at 0 °C under constant stirring and the mixture was stirred for a further 2 h at room temperature. The resulting mixture was washed with a saturated solution of NaHCO_3_ (2 × 20 mL) and brine, then dried (MgSO_4_). The solvent was removed *in vacuo* and the crude product was purified by column chromatography (silica gel, hexane: ethyl acetate gradient) to give ester (**3**) (1.29 g, 3.55 mmol, 81% yield). ^1^H NMR (400 MHz; CDCl_3_; Me_4_Si) δ_H_: 0.85 (3 H, t, *J* = 1.6 Hz, CH_2_C*H*_3_), 0.95–1.04 (7 H, m, CH(C*H*_3_)_2_ and C*H*(CH_3_)_2_), 1.05 (3 H, d, *J* = 7.0 Hz, CHC*H*_3_), 1.35–1.45 (1H, m, C*H*_2a_CH_3_), 1.54–1.64 (1H, m, C*H*_2b_CH_3_), 2.10 (2 H, t, *J* = 2.0 Hz, COC*H*_2_), 2.22–2.33 (1H, m, C*H*CH_3_), 4.36 (1H, dd, *J* = 7.0 and 11.0 Hz, OC*H*_2a_CH), 4.50 (1H, dd, *J* = 3.5 and 11.0 Hz, OC*H*_2b_CH), 4.90–4.93 (1H, m, NHC*H*), 5.18 (2 H, q, *J =* 6.0 Hz, C*H*_2_-Ar), 6.22 (1H, br d, *J =* 7.3 Hz, NH), *7*.38–7.31 (5 H, m, Ar*H*). ^13^C NMR (400 MHz; CDCl_3_; Me_4_Si) δ_C_: 11.5 (CH_2_*C*H_3_), 16.5 (CH*C*H_3_), 22.4 (CH(*C*H_3_)_2_), 22.5 (CH(*C*H_3_)_2_), 26.2 (*C*H_2_CH_3_), 26.6 (*C*H(CH_3_)_2_), 40.8 (*C*HCH_3_), 45.8 (CO*C*H_2_CH(CH_3_)_2_), 52.0 (NH*C*H), 63.8 (O*C*H_2_CH), 67.6 (*C*H_2_-Ar), 128.4 (*C*H-Ar), 128.6 (*C*H-Ar), 128.7 (*C*H-Ar), 135.0 (*C*-Ar), 169.5 (NHCH*C*OO), 172.3 (NH*C*O), 176.3 (CH_3_CH_2_CH(CH_3_)*C*OO). HRMS (ESI^+^): Found (MH^+^): 364.2118, C_20_H_29_NO_5_ requires 364.2119.

#### *N*-3-Methylbutanoyl-*O*-(*S*)-2-methylbutanoyl-L-serine (**4**)

To a stirred solution of ester (**3**) (1.26 g, 3.47 mmol) in dry ethanol (50 mL), was added 10% Pd-C catalyst (280 mg) at room temperature. The mixture was then stirred for 24 h under H_2_, after which time the catalyst was removed by filtration. The solvent was removed *in vacuo* to give the final product (**4**) (0.85 g, 3.11 mmol, 90% yield). ^1^H NMR (400 MHz; CDCl_3_; Me_4_Si) δ_H_: 0.89 (3 H, t, *J* = 7.5 Hz, CH_2_C*H*_3_), 0.95-1.00 (7 H, m, CH(C*H*_3_)_2_ and C*H*(CH_3_)_2_), 1.13 (3 H, d, *J* = 7.0 Hz, CHC*H*_3_), 1.41–1.52 (1H, m, C*H*_2a_CH_3_), 1.61–1.71 (1H, m, C*H*_2b_CH_3_), 2.12 (2 H, d, *J* = 2.2 Hz, COC*H*_2_), 2.40 (1H, sext., *J =* 7.0 Hz, C*H*CH_3_), 4.43 (1H, dd, *J* = 3.5 and 11.5 Hz, OC*H*_2a_CH), 4.55 (1H, dd, *J* = 5.0 and 11.5 Hz, OC*H*_2b_CH), 4.82–4.87 (1H, m, NHC*H*), 6.35 (1H, br d, *J =* 8.0 Hz, N*H*). ^13^C NMR (400 MHz; CDCl_3_; Me_4_Si) δ_C_: 11.4 (CH_2_*C*H_3_), 16.3 (CH*C*H_3_), 22.3 (CH_2_CH(*C*H_3_)_2_), 22.4 (CH_2_CH(*C*H_3_)_2_), 26.2 (*C*H_2_CH_3_), 26.6 (CH_2_*C*H(CH_3_)_2_), 40.9 (*C*HCH_3_), 45.7 (CO*C*H_2_CH(CH_3_)_2_), 51.0 (NH*C*H), 63.6 (O*C*H_2_CH), 173.3 (NH*C*O), 176.6 (CH_3_CH_2_CH(CH_3_)*C*OO), 207.3 (*C*OOH). HRMS (ESI^+^): Found (MH^+^): 274.1649, C_13_H_23_NO_5_ requires 274.1649.

### Laboratory bioassays

*Y-tube testing*. Initial bioassays were conducted following a method similar to those previously reported^22^. Briefly, male spiders upon reaching sexually maturity (identified by their pedipalp sclerites) were introduced into a holding chamber (id = 2 cm, length = 8 cm) on their web at the start of the glass y-tube (id = 2 cm, length of each arm = 19 cm) containing bamboo skewers for the spiders to walk on. Each arm of the y-tube had a different treatment attached via a small cylindrical glass jointed chamber. Treatments were virgin spider silk (10 webs wrapped around a small wooden ‘V’), or a wooden ‘V’ control. Humidified charcoal filtered air was pushed into each arm using at a flow rate of 100 mL/min. The male spider was given a maximum of 30 min to make a decision, and his first choice was recorded. Each spider was used for only one replicate. All glassware was washed with Pyroneg™, rinsed with water and oven baked (3 h at 150 °C) between each replicate and new bamboo skewers were use for each replicate. Treatments were randomly assigned to each y-tube arm for each replicate.

*Still-air arena testing*. Male spiders on their web, were placed in the centre of a 44 cm x 31 cm rectangular enclosed still air arena, containing two odour sources, with each source on a different side of the arena, placed 12 cm from the edge of the arena along the centre line. Three different trials were run, using an odour source vs. a control and 10 different male spiders were used for each trial. Trial 1: The odour sources tested were virgin female silk (one web wrapped around a small wooden ‘V’) and a wooden ‘V’ control. Trial 2: a solvent control (100 µL of DCM loaded into a 20 mm L × 10 mm D red rubber septum, supplied by West Pharmaceutical Services, Australia) and a synthetic mixture of the pheromone candidates (0.11 mg of 2-methylpropanoic acid, 0.29 mg of (*S*)-2-methylbutanoic acid, and 0.30 mg of 2-pyrrolidone in 100 µL of DCM loaded into a red rubber septum). Trial 3: The above synthetic mixture plus 1 mg of *N*-3-methylbutanoyl-*O*-(*S*)-2-methylbutanoyl-L-serine in 16 µL of DCM applied to the outside of the septum vs. a solvent control. The short-range cue was applied by slowly dispensing the solution from a pipette to the outside of the septum so as to leave a thin film on top of the septum. The spiders were given a maximum of 30 min to select a treatment, and their first choice was recorded. The arena was lined with clean aluminium foil, which was changed between every replicate. The ratio of compounds used in the septum was chosen based on the average peak areas from the chromatograms of the virgin silk while giving consideration to the relative volatilities of the different compounds. The loading of compounds used in the septum released a similar level of the volatiles to the virgin silk when measured by SPME (unpublished data). Treatments were randomly assigned to each side of the arena for each replicate.

### Field trapping trials

Two field trapping trials were conducted at three Cromwell vineyard sites known to be inhabited by redback spiders (Site 1 = latitude − 44.949° longitude 169.226°, Site 2 = latitude − 44.954° longitude 169.256° and Site 3 = latitude − 45.003° longitude 169.207°). Without specialised spider traps available, we chose the common delta design insect traps (28 cm L × 20 cm W × 13 cm H supplied by UPL New Zealand Ltd) with white sticky bases (19 cm L × 18 cm W supplied by UPL New Zealand Ltd) for the trapping trials. Red coloured traps were used to reduce insect by-catch^26^. The traps were staked onto the ground under the vines using four tent pegs per trap to hold them in place. The first trial/sampling period from 16 January 2025 to 30 January 2025 used the polybutene glue of the sticky base as a dispenser. Two treatment types were tested; a solvent control (200 µL of DCM) and a synthetic pheromone mixture (1.9 mg of 2-methylpropanoic acid, 5.1 mg of (*S*)-2-methylbutanoic acid, 3.0 mg of 2-pyrrolidone and 1 mg of *N*-3-methylbutanoyl-*O*-(*S*)-2-methylbutanoyl-L-serine (**4**) made up to a total volume of 200 µL with DCM), where each treatment was dissolved in the sticky base glue. The second trial/sampling period from 1 February 2025 to 13 February 2025, tested another dispenser type, red rubber septa, like those used for the bioassays. Here the DCM solvent/synthetic mixture was added to the well of the septa, with the DCM carrying the compounds into the rubber matrix. The loading of compounds was decreased for the septa (compared to the glue) down to 1.3 mg of 2-methylpropanoic acid, 3.6 mg of (*S*)-2-methylbutanoic acid, and 2.1 mg of 2-pyrrolidone due to the reduced solubility of the compounds in the septa. The short-range cue, *N*-3-methylbutanoyl-*O*-(*S*)-2-methylbutanoyl-L-serine (**4**) was kept at the same loading (1 mg) and applied to the outside of the septa in a volume of 16 µL of DCM as described above. Each vineyard site had five replicates of solvent control and synthetic pheromone mix put out in pairs across the site.

### Statistical analyses

All statistical analyses were performed using R version 4.5.0 (R Core Team 2025) software^27^. The results from the two laboratory bioassays were analysed using chi-square tests. For the Y-tube and still-air arena experiments, we tested whether the three outcomes of no response, stimulant (either virgin silk, volatile synthetic compounds or volatile plus contact synthetic compounds) and control differed significantly and whether the two responses (stimulant and control) differed. For the field trials we used a GLM with a quasipoisson error distribution to analyse differences between sampling periods, sites and treatments (all blank lures and all pheromone lures).

## Results

### Silk analysis and compound identification

**Fig. 2:**
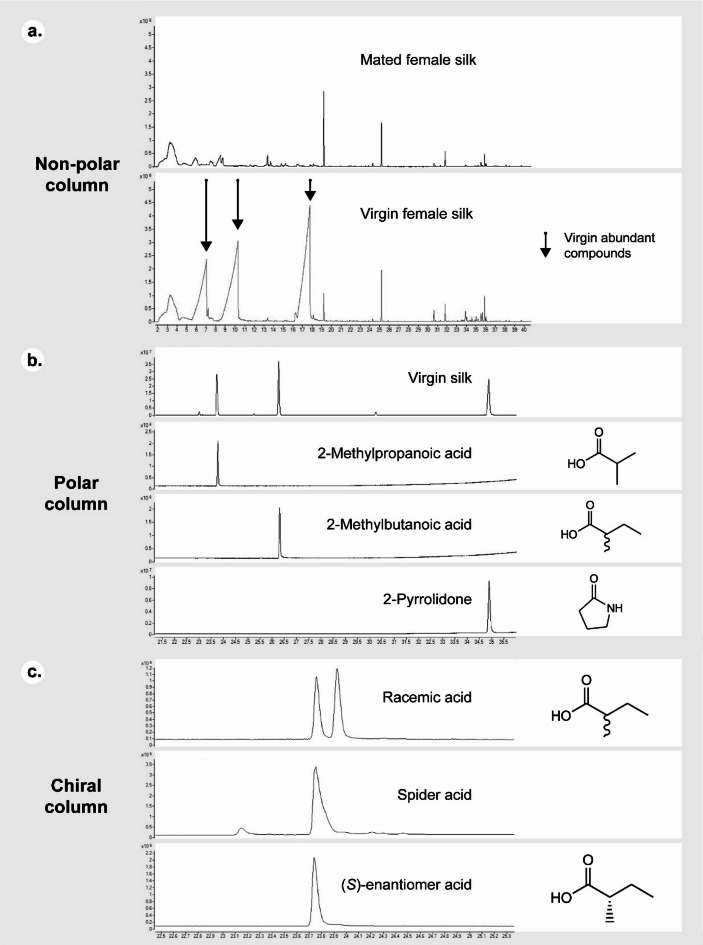
GC-MS analysis of silk volatiles and synthetic standards on different column types to identify potential air-borne attractants released from the silk of virgin redback female spiders. (**a**) Chromatograms of volatiles from mated versus virgin female redback spider silk on a DB-5ms UI non-polar column. (**b**) Chromatograms of virgin female redback spider silk versus synthetic standards on a DB-wax polar column. (**c**) Chromatograms of racemic 2-methylbutanoic acid versus virgin female redback spider silk versus (S)-2-methylbutanoic acid on a Cyclosil B chiral column.

Initial analysis of silk headspace volatiles on the non-polar column showed three abundant virgin compounds (Fig. 2a) and the broad peak shape implied the compounds were polar in nature. MS fragmentation and NIST library comparison suggested the compounds were 2-methylpropanoic acid, 2-methylbutanoic acid, and 2-pyrrolidone. To confirm the identity of these compounds, solvent extracts of the virgin female silk were used. Comparison with solutions of synthetic standards indicated the identifications were correct. Further analysis on a polar column confirmed the identities of 2-methylpropanoic acid and 2-pyrrolidone (Fig. 2b). 2-Methylbutanoic acid could exist as two possible enantiomers, so the extract was run on a chiral column which showed that the virgin female spider produced the (*S*)-enantiomer only (Fig. 2c).

**Fig. 3:**
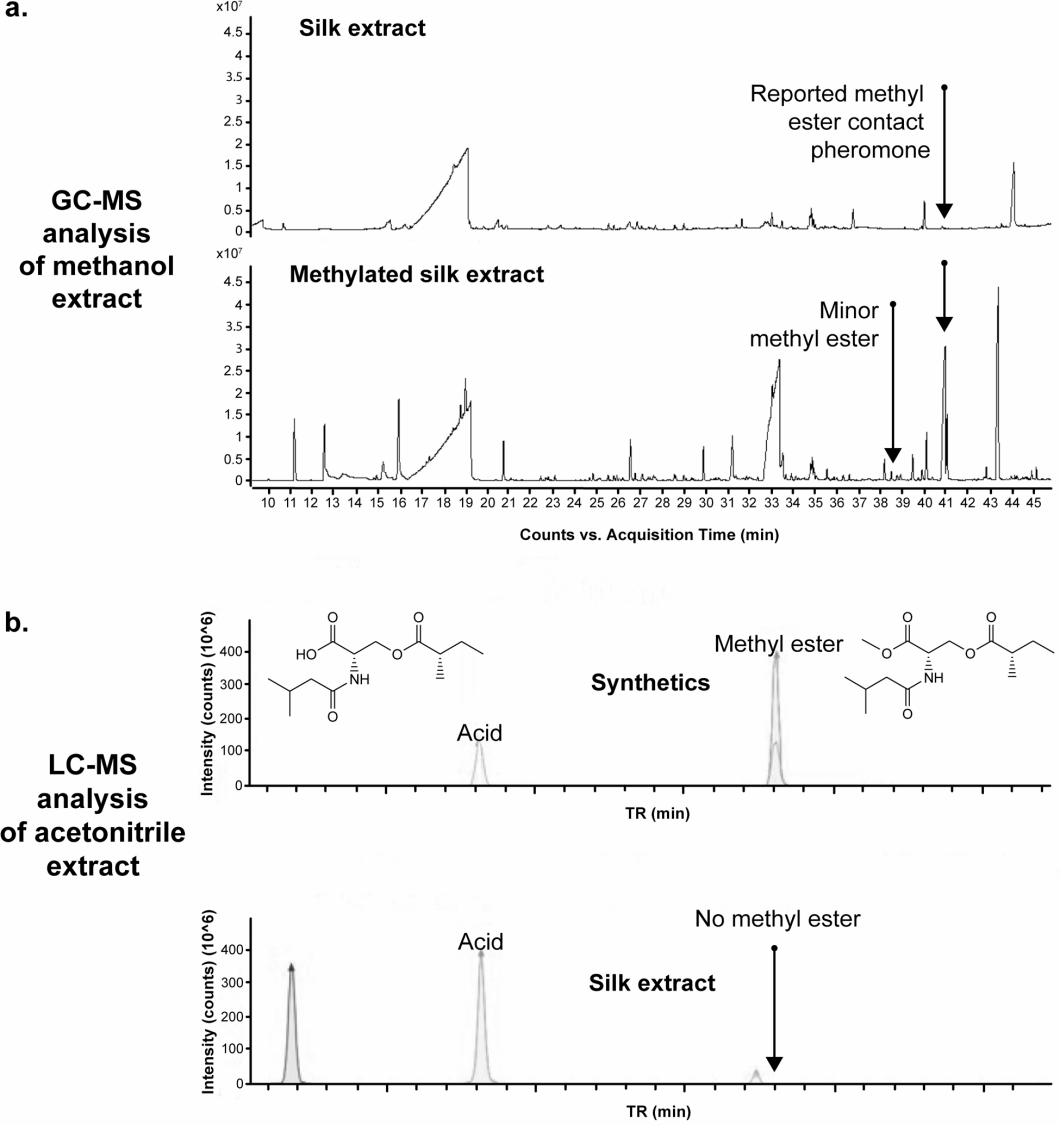
GC-MS (**a**) and LC-MS (**b**) analysis of silk extracts to confirm the identity of the potential short-range attractant of the Australian redback spider. (**a**) Chromatograms from GC-MS analysis of methanol virgin redback spider silk extract versus the same extract following laboratory microscale methylation. (**b**) Chromatograms from LC-MS analysis of acetonitrile silk extract (bottom) versus synthetic contact pheromone candidates (top).

Due to the high abundance of the short-chain acids in the silk extract, large amounts of the previously reported contact pheromone *N*-3-methylbutanoyl-*O*-(*S*)-2-methylbutanoyl-L-serine methyl ester (**11**) were expected to be visible in the chromatograms too, but only trace amounts were present in the 24 h methanol extracts (Fig. 3a). The silk extract was then methylated to convert any possible acid compounds to the related methyl esters for easier visualization on the GC-MS. Following methylation, large amounts of the previously reported contact pheromone and its related minor component *N*-3-methylbutanoyl-*O*-methylpropanoyl-L-serine methyl ester (**11**) were visible (Fig. 3a). This suggested that the small amount present in the initial methanol extract had come from methylation of the related acid in the extract. Therefore, fresh virgin silk was extracted with acetonitrile to avoid any unintended methylation of the extract by the solvent and the solution was analysed by LC-MS (Fig. 3b). Here the candidate contact pheromone components were shown to be the carboxylic acids rather than the previously reported methyl esters (Fig. 3b). The compounds found in the acetonitrile extract of the redback silk were *N*-3-methylbutanoyl-*O*-(*S*)-2-methylbutanoyl-L-serine (**4**) and *N*-3-methylbutanoyl-*O*-2-methylpropanoyl-L-serine (**8**). None of the previous reported major contact pheromone component *N*-3-methylbutanoyl-*O*-(*S*)-2-methylbutanoyl-L-serine methyl ester (**11**) and minor component *N*-3-methylbutanoyl-*O*-methylpropanoyl-L-serine methyl ester were found in the acetonitrile extract.

### Laboratory bioassays

**Fig. 4:**
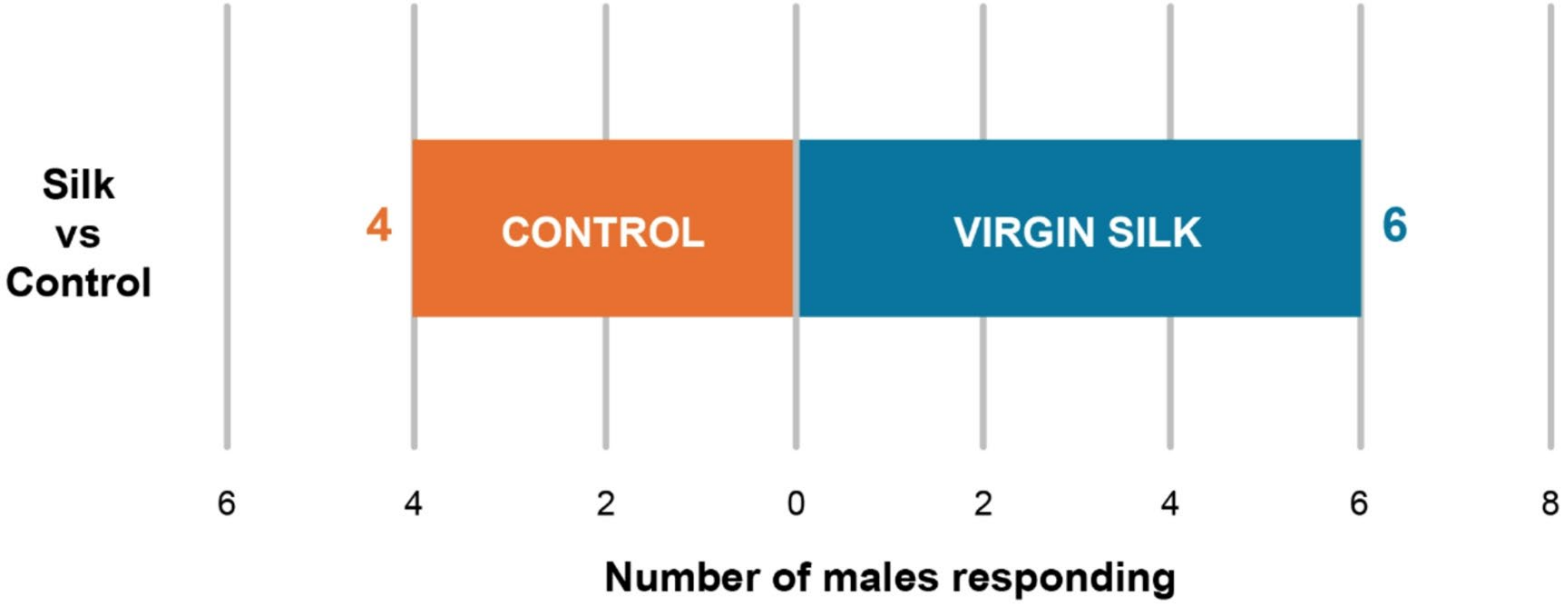
Y-tube testing virgin redback male spiders (*n*=41individuals) response to virgin female redback silk versus an empty silk frame, 31 out of the 41 male spiders tested gave no response.

*Y-tube testing*. Initial testing using virgin silk versus an empty wooden ‘V’ silk frame proved ineffective, with only 10 out of the 41 males tested giving a response (Fig. 4), which was not significantly different (χ^2^ = 0.40, df = 1, *p* = 0.53).

**Fig. 5:**
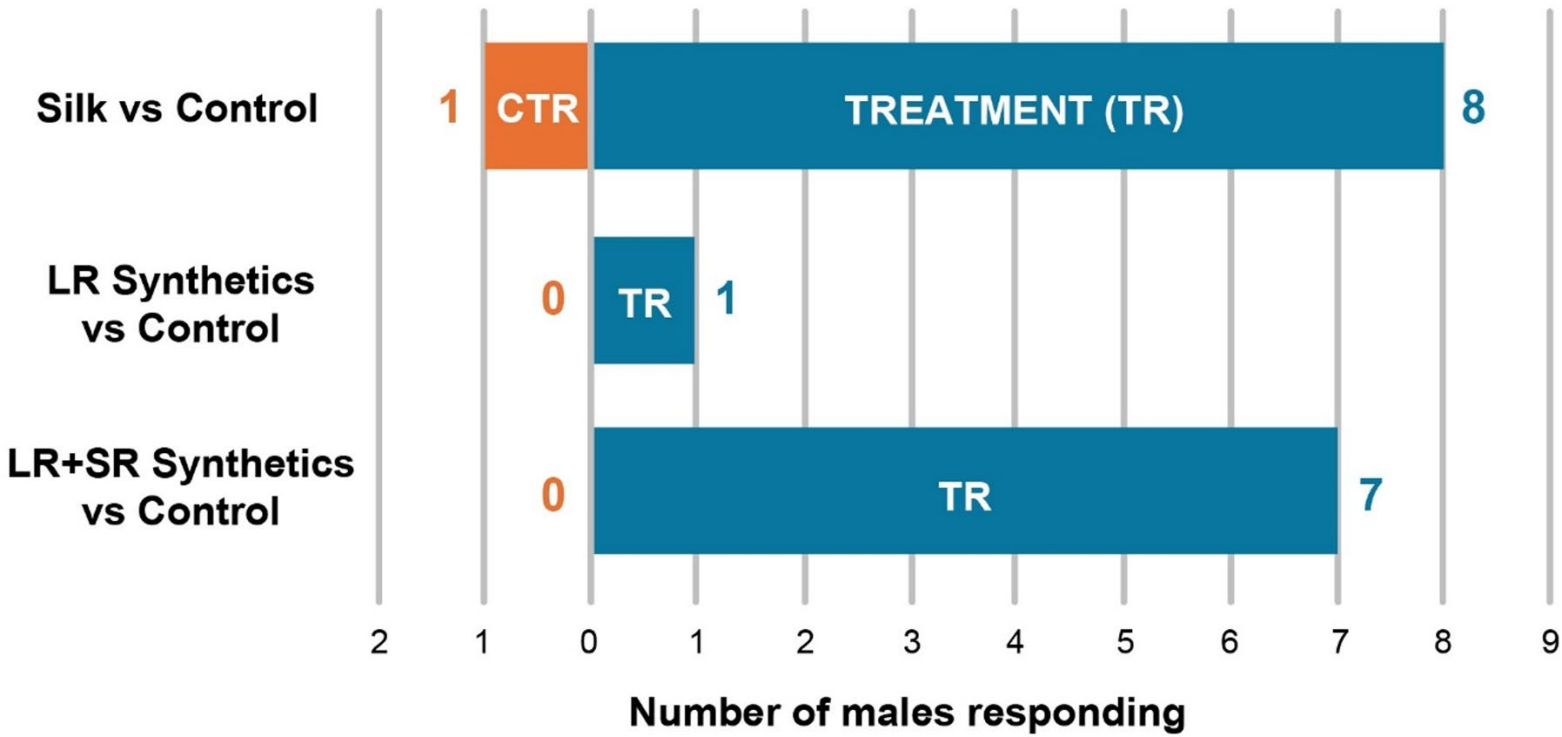
Still-air bioassays testing response of virgin male redback spiders (*n* = 10 for each treatment and control pair) to virgin silk versus control (top), candidate long-range (LR) synthetic attractants versus solvent control (middle) and candidate LR attractants plus candidate short-range (SR) synthetic attractant versus solvent control (bottom).

*Still-air arena testing*. Initial testing focussed on the response of males (*n* = 10) to virgin silk versus a blank control (Fig. 5). Of the 10 males tested eight went to the virgin silk, one went to the control and one did not respond (χ^2^ = 9.80, df = 2, *p* = 0.0074). Males were significantly non-responsive to the long-range synthetic candidates (χ^2^ = 14.61, df = 2, *p* = 0.0007), with only one male contacting the synthetic lure, the nine other males were non-responsive (Fig. 5). When the contact/short-range synthetic compound was added to the airborne compounds the males were attracted to the treated septum, with seven out of the 10 males tested contacting the synthetic lure, while none of the males went to the solvent control (Fig. 5) (χ^2^ = 7.40, df = 2, *p* = 0.025).

### Field trapping trials

**Fig. 6:**
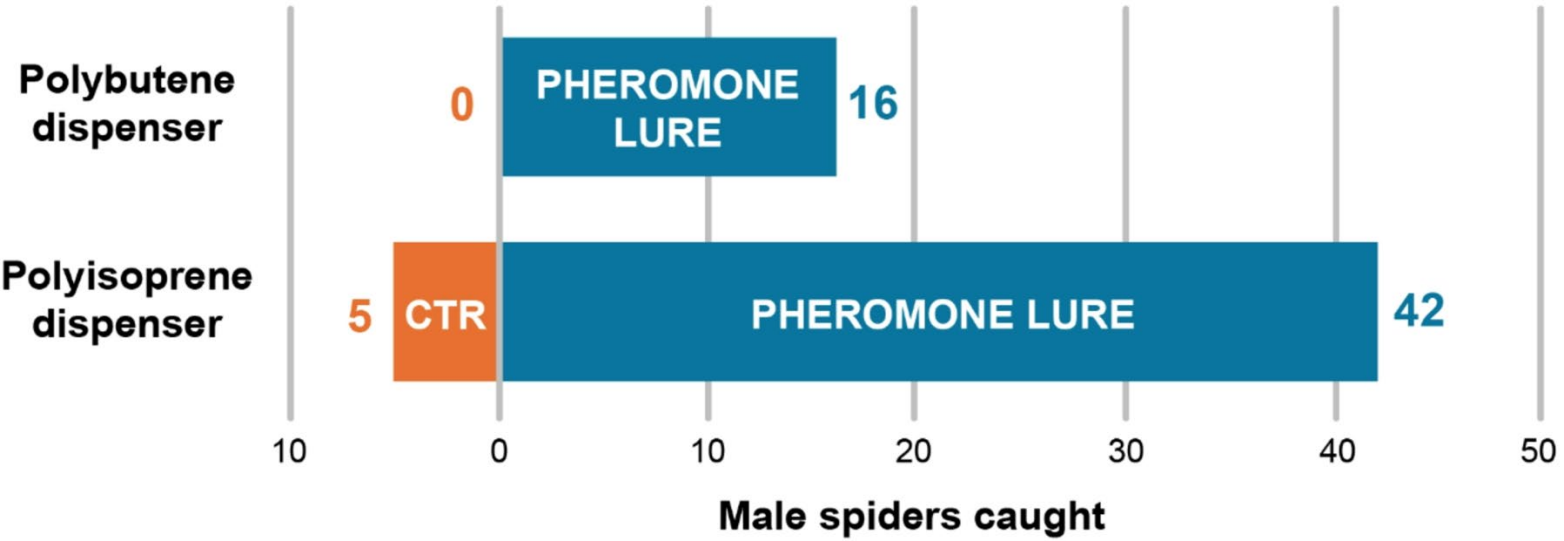
Capture of male redback spiders to synthetic pheromone versus control traps in Cromwell vineyards 2025. Polybutene dispenser trial, sampling period one (top) and polyisoprene dispenser trial, sampling period two (bottom).

Trapping trials using both dispenser types successfully caught male redback spiders (Fig. 6). The polybutene glue on the sticky bases loaded with the pheromone candidates caught a total of 16 male redback spiders over the two-week trapping period in late January, while the control traps caught no male spiders (Fig. 6). The polybutene glue appeared to lose the odour very quickly and was completely depleted after two weeks in the field (unpublished data). The red rubber septa (polyisoprene) dispensers loaded with the pheromone candidates caught a total of 42 male redback spiders while the solvent control septa caught a total of five male spiders over the second two-week trapping period (Fig. 6) in early February. The polyisoprene dispensers released the pheromone candidates more slowly and were still releasing pheromone after two weeks in the field (unpublished data). The pheromone lures attracted significantly more spiders than the control treatment (*p* = 5.186 × 10^− 5^, F = 19.2758, df = 1,58), while almost three times more spiders were attracted during the second sampling period than in the first sampling period, (*p* = 0.0188, F = 19.2758, df = 1,55). There were no consistent significant differences in counts among the sites (*p* = 0.3242, F = 1.1498, df = 1,56).

## Discussion

Our results showed that the two abundant carboxylic acids in the headspace of the virgin female redback spider silk were 2-methylpropanoic acid and (*S*)-2-methylbutanoic acid rather than the previously reported respective constitutional isomers butanoic acid and 3-methylbutanoic acid^21^. The identification of 2-methylpropanoic acid and (*S*)-2-methylbutanoic acid as redback spider pheromone components was confirmed on three GC column types in comparison with synthetic standards (Fig. 1) and is in line with the research of other Theridiidae pheromones^20,22,24^. For the related false widow, it was shown that the airborne pheromone attractants; butanoic acid, 2-methylpropanoic acid and hexanoic acid were the hydrolysis products of the respective contact pheromone components; *N*-4-methylpentanoyl-*O*-butanoyl-L-serine, *N*-4-methylpentanoyl-*O*-2-methylpropanoyl-L-serine, and *N*-4-methylpentanoyl-*O*-hexanoyl-L-serine^22^. Similar serine derivatives and their related hydrolysis products have also been shown to be pheromones for the western black widow^24^. If the redback spider employed a similar hydrolysis of the previously reported contact pheromone compounds *N*-3-methylbutanoyl-*O*-(S)-2-methylbutanoyl-L-serine methyl ester (**11**) and *N*-3-methylbutanoyl-*O*-2-methylpropanoyl-L-serine methyl ester^20^, then the airborne pheromone compounds would be expected to include (*S*)-2-methylbutanoic acid and 2-methylpropanoic acid, which is what we found in the headspace of the virgin female redback silk. An abundance of the amide 2-pyrrolidone was also found in the headspace which was similar to the solvent extracts of Jerhot et al. (2010) where this compound was abundant too. The origin of the 2-pyrrolidone has not been established for the Theridiidae spiders, but it is possibly a further break down product of the contact pheromones due to its abundance on the virgin female silk compared to the mated female silk. Unexpectedly only trace amounts of the previously reported contact pheromone compound *N*-3-methylbutanoyl-*O*-(*S*)-2-methylbutanoyl-L-serine methyl ester (**11**) could be found in our methanol silk extract. This was surprising as the methyl ester had been abundant in previously reported extracts^20^ but was barely detectable in ours. Considering this, we noted that other Theridiidae contact pheromones have recently been identified as carboxylic acids rather than the methyl esters using LC-MS which is better suited to the identification of polar compounds than GC-MS^22–24^. This led us to consider that the contact pheromone components of the redback spider may in fact be the related carboxylic acids rather than the methyl esters previously described, whereby the spider compounds may have undergone serendipitous methylation in the extraction solvent methanol. This was supported by methylation of the silk extract, which when treated with acidified methanol, showed an abundance of the methyl ester (**11**) (Fig. 3). This was confirmed with new silk extracts using acetonitrile as the solvent instead of methanol. Acetonitrile extracts showed none of the previously reported methyl ester (**11**) but an abundance of the related carboxylic acid *N*-3-methylbutanoyl-*O*-(*S*)-2-methylbutanoyl-L-serine (**4**), which was confirmed by comparison to a synthetic standard via LC-MS (Fig. 2).

Laboratory-based bioassays to test these candidate pheromone compounds proved difficult and the spiders did not behave naturally in the initial setups. Y-tube bioassays similar to those reported in the literature^22^ were initially used for behavioural testing, but these gave very poor response rates with most of the male spiders preferring to sit in their own web rather than move into the arms of the y-tube (Fig. 4). No preference was seen to the webs from virgin females compared to empty chambers, with those males that did move off their web appearing to just sprint randomly down one arm or the other, and no typical mate-finding behaviour such as leg waving was observed. This led us to pursue other methods with the male spiders appearing much more comfortable in the larger still-air bioassay arena. Here the male spiders roamed freely off their webs compared to their either stationary or sprinting behaviour in the y-tube. With the initial success of the still-air arena we expected the airborne synthetic pheromone blend to be a success, but only one out of the 10 males tested responded. What this isolated result did not show, was that several of the male spiders appeared to be attracted to the septa treated with the synthetics as they changed course and walked directly towards it only to stop when a few centimetres away, then after a long pause they moved away. It was this arrestant behaviour that suggested they needed a short-range cue as well for the spiders to actually make contact with the treated septa. Addition of the contact pheromone candidate immediately improved the attraction rate to 70% giving us confidence that our lure was attractive to the male spiders.

Field trapping spiders with synthetic pheromones appears to be very rare, with trials from only two species reported in the last ten years, both occurring in the family Theridiidae^22,24^. For the false widow a total of only nine male spiders were captured over the four-month trial period^22^ while synthetic pheromone trapping of western black widow spiders caught a total of six male spiders^24^. Both of these trials used perforated\open vials containing the carboxylic acids dispersed in mineral oil as a dispenser. The traps used in the false widow trial were sticky bases while the western black widow trial used fake webs to retain the spiders. Carboxylic acids have been known to have difficulties releasing from standard dispensers types such as rubber septa^28^, hence for our first field trial we tested the polybutene glue from the sticky base as a release medium. The glue was also chosen as a dispenser to provide a large surface area for release of the pheromone, similar in size to a web. While the glue dispenser was successful, capturing 16 male spiders over the two-week test period, it was difficult to handle and the compounds were released from it too quickly, being completely depleted at the end of the two weeks (unpublished data). For the second trial, we decided to try the polyisoprene rubber septa as they had been used successfully in the bioassay trials. Over the two-week trial period the polyisoprene septa proved surprisingly effective catching 42 male spiders and were still releasing compounds after the two weeks in the field, representing a promising candidate as an initial dispenser for the redback spider compounds. For both field trials the loading of 2-methylpropanoic acid and (*S*)-2-methylbutanoic acid was increased relative to the 2-pyrrolidone and contact pheromone component in comparison with the bioassay trials to try to improve the longevity of the lures based on the higher volatility of these two short chain acids.

More work is required to optimise the spider trapping system. In both field trials some of the male spiders were not caught in the traps but had built webs above the sticky base of the traps, hence they may have chased off other male spiders coming to the lure and potentially lowered trap catch. Further work on trap type and design is required to reduce this behaviour. Based on the bioassay observations, the contact pheromone component was included in the lure as we thought it may be an important cue for getting the final close-range attraction/contact with the trap. The improved trap catch in our trials compared to previous spider trapping experiments^22,24^ may be a result of the inclusion of the contact pheromone in the lure, although other factors such as dispenser type and/or population density could also be attributing factors, this also needs further testing. Another notable mention was that while all virgin female silk headspace samples contained the three airborne compounds; 2-methylpropanoic acid, (*S*)-2-methylbutanoic acid and 2-pyrrolidone, their ratio varied substantially between the samples. Across the headspace samples taken, each of these volatiles was the largest peak in at least one of the headspace replicates. Our long silk sampling time of up to four weeks may have played a role in this but with virgin females all of similar age and fed on similar diets no obvious explanation exists. Further work is required to try and understand this ratio and optimise it for trap efficacy.

## Conclusions

The pheromone candidates identified from the silk of virgin female *L. hasseltii* were successfully used to capture males of the species and represent the first step in the development of a pest management/surveillance tool for these invasive spiders.

## Supplementary Information

Below is the link to the electronic supplementary material.

Supplementary Material 1

## Data Availability

The datasets generated during and/or analysed during the current study are available from the corresponding author on reasonable request.
