# Correction: Identification and testing of sex pheromone components of the invasive Australian redback spider (*Latrodectus hasseltii*)

**DOI:** 10.1038/s41598-026-61187-1

**Published:** 2026-07-07

**Authors:** Andrew M. Twidle, Thomas E. S. Sullivan, Meikura T. Arahanga, Lisa I. Pilkington, Devon T. Bryant, Nigel I. Joyce, Nicola J. Sullivan, Tara J. Murray, Cor J. Vink

**Affiliations:** 1 New Zealand Institute for Bioeconomy Science Limited, Christchurch Mail Centre, Private Bag 4704, Christchurch, 8140 New Zealand; 2 Bioprotection Aotearoa, Lincoln University, PO Box 85084, Lincoln, 7647 New Zealand; 3 School of Chemical Sciences, University of Auckland, Private Bag 92019, Auckland, New Zealand; 4 Te Pūnaha Matatini, Auckland, 1142 New Zealand; 5 Department of Pest-Management and Conservation, Lincoln University, PO Box 85084, Lincoln, 7647 New Zealand; 6 Department of Conservation (DOC), PO Box 5244, Dunedin, 9054 New Zealand

Correction to: *Scientific Reports* 10.1038/s41598-026-49837-w, published online 27 April 2026

The original version of this Article contained errors in the Title of the paper and in the title of the Supplementary Material. Specifically, the scientific name of the redback spider was erroneously given as *Lactrodectus hasseltii* when the correct scientific is *Latrodectus hasseltii*. As a result, the title was updated from:

“Identification and testing of sex pheromone components of the invasive Australian redback spider (*Lactrodectus hasseltii*).”

to now read:

“Identification and testing of sex pheromone components of the invasive Australian redback spider (*Latrodectus hasseltii*).”

The same error occurred in the title of the Supplementary Material.

The original Article and Supplementary Material have been corrected.

