# Supplementary material for: Identification and testing of sex pheromone components of the invasive Australian redback spider (Latrodectus hasseltii)
Source: Sci Rep. 2026 Apr 27;16:19448. doi: 10.1038/s41598-026-49837-w (PMC13287630; doi:10.1038/s41598-026-49837-w)
Supplement: Supplementary file 1 — Supplementary Material 1 [file 41598_2026_49837_MOESM1_ESM.pdf]

Supporting information

Identification and testing of sex pheromone components of the invasive Australian redback spider (*Latrodectus hasseltii*)

Andrew M Twidle,[1*] Thomas ES Sullivan,[1] Meikura T Arahanga,[2] Lisa I Pilkington,[3,4] Devon T Bryant,[3] Nigel I Joyce,[1] Nicola J Sullivan,[1,5] Tara J Murray,[6] Cor J Vink[5]

[1] *New Zealand Institute for Bioeconomy Science Limited, Private Bag 4704, Christchurch Mail Centre, Christchurch 8140, New Zealand*

[2] *Bioprotection Aotearoa, PO Box 85084, Lincoln University, Lincoln 7647, New Zealand*

[3] *School of Chemical Sciences, University of Auckland, Private Bag 92019 Auckland, New Zealand*

[4] *Te Pūnaha Matatini, Auckland 1142, New Zealand*

[5] *Department of Pest-Management and Conservation, PO Box 85084, Lincoln University, Lincoln 7647, New Zealand*

[6] *Department of Conservation (DOC), PO Box 5244, Dunedin 9054, New Zealand*

*Corresponding author: andrew.twidle@plantandfood.co.nz

Andrew Twidle
orcid 0000-0001-6294-5526

Thomas Sullivan
orcid 0000-0002-1606-9753

Lisa Pilkington
orcid 0000-0002-9292-3261

Devon Bryant
orcid 0000-0002-2409-5396

Nigel Joyce
orcid 0000-0002-1451-4841

Nicola Sullivan
orcid 0000-0001-8014-9945

Tara Murray
orcid 0000-0001-8332-5406

Cor Vink
orcid 0000-0003-4237-0117

General procedure for acid chloride synthesis
3-Methylbutanoyl chloride was prepared by reaction of neat 3-methylbutanoic acid with neat thionyl chloride (2eq) under argon, taking the reaction mixture from room temperature to reflux. After 2 h at reflux the resulting mixture was cooled, then purified under high vacuum to give the desired acid chloride which was used without further purification.

Synthetic procedures for previously reported compounds

N-Boc-*O*-2-methylpropanoyl-L-serine benzyl ester (**5**).

To a stirred solution of *N*-Boc-L-serine benzyl ester (500 mg, 1.69 mmol) in dry dichloromethane (DCM) (20 mL) at 0°C, 2-methylpropanoic acid (164 mg, 1.86 mmol) was added. This was followed by the addition of 4-dimethylaminopyridine (41 mg, 0.34 mmol) and dicyclohexylcarbodiimide (416 mg, 2.02 mmol). The resulting mixture was allowed to warm to room temperature, then stirred for 48 h. Upon completion the dicyclohexylurea precipitate was filtered off and the filtrate was washed with a saturated solution of $NaHCO_3$ (20 mL) then dried ($MgSO_4$). The solvent was removed *in vacuo* and the crude product was purified by column chromatography (silica gel, hexane: ethyl acetate gradient) to give ester (**5**) (428 mg, 1.17 mmol, 69% yield). ^{1}H NMR (400 MHz; $CDCl_3$; Me_4Si) δ_H: 1.08 (6H, dd, *J* = 7.0 and 8.5 Hz, CH(C$\underline{H}_3$)$_2$), 1.44 (9H, s, C(CH_3)$_3$), 2.47 (1H, sept., *J* = 7.0 Hz, C$\underline{H}$(CH_3)$_2$), 4.30 (1H, dd, *J* = 3.5 and 11.3 Hz, OC$\underline{H}_{2a}$CH), 4.47 (1H, dd, *J* = 4.0 and 11.3 Hz, OC$\underline{H}_{2b}$CH), 4.60-4.64 (1H, m, NHC$\underline{H}$), 5.18 (2H, d, *J* = 13.4 Hz, C$\underline{H}_2$-Ar), 5.28 (1H, br d, *J* = 8.0 Hz,N$\underline{H}$) 7.30-7.38 (5H, m, Ar$\underline{H}$). ^{13}C NMR (400 MHz; $CDCl_3$; Me_4Si) δ_C: 18.7 (CH($\underline{C}H_3$)$_2$), 18.8 (CH($\underline{C}H_3$)$_2$), 28.3 (C($\underline{C}H_3$)$_3$), 33.8 ($\underline{C}$H(CH_3)$_2$), 53.1 (NH$\underline{C}$H), 64.1 (O$\underline{C}H_2$CH), 67.6 (Ar-$\underline{C}H_2$), 77.2 ($\underline{C}$(CH_3)$_3$), 128.3 ($\underline{C}$H-Ar), 128.5 ($\underline{C}$H-Ar), 128.6 ($\underline{C}$H-Ar), 135.1 ($\underline{C}$-Ar), 169.8 (NHCH$\underline{C}$OO), 172.8 (NH$\underline{C}$OO), 176.5 ((CH_3)$_2$CH$\underline{C}$OO). HRMS (ESI$^+$): Found (MH$^+$): 366.1911, $C_{19}H_{27}NO_6$ requires 366.1911

N-Hydro-*O*-2-methylpropanoyl-L-serine benzyl ester (**6**).

To a stirred solution of ester (**5**) (371 mg, 1.02 mmol) in dry DCM (20 mL), trifluoroacetic acid (5 mL) was added dropwise at room temperature. After 2 h of mixing, the solvent, remaining acid and other volatiles were removed *in vacuo*. The crude amino ester (**6**) was then characterized by NMR and used directly in the next step without purification. ^{1}H NMR (400 MHz; $CDCl_3$; Me_4Si) δ_H: 1.02-1.07 (6H, m, CH(C$\underline{H}_3$)$_2$), 2.42-2.50 (1H, m, C$\underline{H}$(CH_3)$_2$), 4.36-4.40 (1H, m, NH_2C$\underline{H}$), 4.51-4.60 (2H, m, OC$\underline{H}_2$CH), 5.15-5.28 (2H, m, C$\underline{H}_2$-Ar), 7.30-7.37 (5H, m, Ar$\underline{H}$), 7.78 (2H, br s, N$\underline{H}_2$). ^{13}C NMR (400 MHz; $CDCl_3$; Me_4Si) δ_C: 18.3 (CH($\underline{C}H_3$)$_2$), 18.5 (CH($\underline{C}H_3$)$_2$), 33.4 ($\underline{C}$H(CH_3)$_2$), 53.0 ($NH_2\underline{C}$H), 61.2 (O$\underline{C}H_2$CH), 69.4 ($\underline{C}H_2$-Ar), 128.6 ($\underline{C}$H-Ar), 128.7 ($\underline{C}$H-Ar), 129.0 ($\underline{C}$H-Ar), 133.9 ($\underline{C}$-Ar), 166.5 (NH_2CH$\underline{C}$OO), 176.7 ((CH_3)$_2$CH$\underline{C}$OO). HRMS (ESI$^+$): Found (MNa$^+$): 288.1206, $C_{14}H_{19}NO_4$ requires 288.1206

N-3-Methylbutanoyl-*O*-2-methylpropanoyl-L-serine benzyl ester (**7**)

Crude amino ester (**6**) (231 mg, 0.87 mmol) was dissolved in dry DCM (20 mL) and triethylamine (361 mg, 3.57 mmol) was added dropwise at 0°C under constant stirring. 3-Methylbutanoyl chloride (307 mg, 2.55 mmol) was then added dropwise at 0°C under constant stirring and the mixture was stirred for a further 2 h at room temperature. The resulting mixture was washed with a saturated solution of $NaHCO_3$ (20 mL x 2) and brine, then dried ($MgSO_4$). The solvent was removed *in vacuo* and the crude product was purified by column chromatography (silica gel, hexane: ethyl acetate gradient) to give ester (**7**) (277 mg, 0.79 mmol, 91% yield). ^{1}H NMR (400 MHz; $CDCl_3$; Me_4Si) δ_H: 0.93-1.00 (7H, m, $COCH_2$CH(C$\underline{H}_3$)$_2$ and $COCH_2$C$\underline{H}$(CH_3)$_2$), 1.08 (6H, dd, *J* = 5.5 and 7.0 Hz, COCH(C$\underline{H}_3$)$_2$), 2.09-2.11 (2H, m, COC$\underline{H}_2$), 2.45 (1H, sept., *J* = 7.0 Hz, COC$\underline{H}$(CH_3)$_2$), 4.34 (1H, dd, *J* = 3.5 and 11.0 Hz, OC$\underline{H}_{2a}$CH), 4.48 (1H, dd, *J* = 4.0 and 11.0 Hz, OC$\underline{H}_{2b}$CH), 4.89-4.93 (1H, m, NHC$\underline{H}$), 5.19 (2H, d, *J* = 9.5 Hz, C$\underline{H}_2$-Ar), 6.22 (1H, br d, *J* = 7.1 Hz, N$\underline{H}$), 7.30-7.38 (5H, m, Ar$\underline{H}$). ^{13}C NMR (400 MHz; $CDCl_3$; Me_4Si) δ_C: 18.4 (COCH($\underline{C}H_3$)$_2$), 18.5 (COCH($\underline{C}H_3$)$_2$), 22.3 (CH_2CH($\underline{C}H_3$)$_2$), 22.4 (CH_2CH($\underline{C}H_3$)$_2$), 26.2 ($CH_2\underline{C}$H(CH_3)$_2$), 34.0 (CO$\underline{C}$H(CH_3)$_2$), 45.9 ($\underline{C}H_2$CH(CH_3)$_2$), 51.8 (NH$\underline{C}$H), 63.7 (O$\underline{C}H_2$CH), 67.6 (Ar-$\underline{C}H_2$), 128.3 ($\underline{C}$H-Ar), 128.6 ($\underline{C}$H-Ar), 128.7 ($\underline{C}$H-Ar), 135.0 ($\underline{C}$-Ar), 169.5 (NHCH$\underline{C}$OO), 172.2 (NH$\underline{C}$OO), 176.7 ((CH_3)$_2$CH$\underline{C}$OO). HRMS (ESI$^+$): Found (MH$^+$): 350.1962, $C_{19}H_{27}NO_5$ requires 350.1968

N-3-Methylbutanoyl-*O*-2-methylpropanoyl-L-serine (**8**)

To a stirred solution of ester (**7**) (245 mg, 0.70 mmol) in dry ethanol (10 mL), 10% Pd-C catalyst (60 mg) was added at room temperature. The mixture was then stirred for 24 h under H_2, after which time the catalyst was removed by filtration. The solvent was removed *in vacuo* to give the final product (**8**) (154 mg, 0.59 mmol, 84% yield). ^{1}H NMR (400 MHz; $CDCl_3$; Me_4Si) δ_H: 0.94-1.00 (7H, m, CH(C$\underline{H}_3$)$_2$ and C$\underline{H}$(CH_3)$_2$), 1.14 (6H, dd, *J* = 1.9 and 7.9 Hz, COCH(C$\underline{H}_3$)$_2$), 2.11-2.15 (2H, m, COC$\underline{H}_2$), 2.51-2.60 (1H, m, COC$\underline{H}$(CH_3)$_2$), 4.41 (1H, dd, *J* = 3.6 and 11.4 Hz OC$\underline{H}_{2a}$CH), 4.48 (1H, dd, *J* = 3.6 and 11.4 Hz, OC$\underline{H}_{2b}$CH), 4.83-4.87 (1H, m, NHC$\underline{H}$), 5.42 (1H, br s, N$\underline{H}$), 6.45 (1H, br s, O$\underline{H}$). ^{13}C NMR (400 MHz; $CDCl_3$; Me_4Si) δ_C: 18.8 (COCH($\underline{C}H_3$)$_2$), 22.3 (CH_2CH($\underline{C}H_3$)$_2$), 22.4 (CH_2CH($\underline{C}H_3$)$_2$), 26.2 ($CH_2\underline{C}$H(CH_3)$_2$), 34.0 (CO$\underline{C}$H(CH_3)$_2$), 45.6 (CO$\underline{C}H_2$CH(CH_3)$_2$), 52.3 (NH$\underline{C}$H), 64.1 (O$\underline{C}H_2$CH), 171.8 ($\underline{C}$OOH), 173.3 (NH$\underline{C}$O), 177.0 ((CH_3)$_2$CH$\underline{C}$OO). HRMS (ESI$^+$): Found (MH$^+$): 260.1492, $C_{12}H_{21}NO_5$ requires 260.1480

N-Boc-*O*-(*S*)-2-methylbutanoyl-L-serine methyl ester (**9**).

To a stirred solution of *N*-Boc-L-serine methyl ester (767 mg, 3.50 mmol) in dry DCM (50 mL) at 0°C, (*S*)-2-methylbutanoic acid (358 mg, 3.51 mmol) was added. This was followed by the addition of 4-

dimethylaminopyridine (89 mg, 0.73 mmol) and dicyclohexylcarbodiimide (722 mg, 3.50 mmol). The resulting mixture was allowed to warm to room temperature, then stirred for 24 h. Upon completion the dicyclohexylurea precipitate was filtered off and the filtrate was washed with a saturated solution of $NaHCO_3$ (25 mL) then dried ($MgSO_4$). The solvent was removed *in vacuo* and the crude product was purified by column chromatography (silica gel, hexane: ethyl acetate gradient) to give ester (**9**) (938 mg, 3.09 mmol, 88% yield). GC-MS data matched those previously reported (Jerhot et al. 2010).

N-Hydro-*O*-(*S*)-2-methylbutanoyl-L-serine methyl ester (**10**).
To a stirred solution of ester (**9**) (193 mg, 0.64 mmol) in dry DCM (15 mL), trifluoroacetic acid (5 mL) was added dropwise at room temperature. After 1 h of mixing, the solvent, remaining acid and other volatiles were removed *in vacuo*. The crude amino ester (**10**) was used directly in the next step without purification.

N-3-Methylbutanoyl-*O*-(*S*)-2-methylbutanoyl-L-serine methyl ester (**11**)
The crude amino ester (**10**) (129 mg, 0.63 mmol) was dissolved in dry DCM (15 mL) and triethylamine (223 mg, 2.20 mmol) was added dropwise at 0°C under constant stirring. 3-Methylbutanoyl chloride (193 mg, 1.60 mmol) was then added dropwise at 0°C under constant stirring and the mixture was stirred for a further 2 h at room temperature. The resulting mixture was washed with a saturated solution of $NaHCO_3$ (2 × 20 mL) and brine, then dried ($MgSO_4$). The solvent was removed *in vacuo* and the crude product was purified by column chromatography (silica gel, hexane: ethyl acetate gradient) to give ester (**11**) (141 mg, 0.49 mmol, 78% yield). GC-MS data matched those previously reported (Jerhot et al. 2010).

Jerhot E, Stoltz JA, Andrade MCB, Schulz S (2010) Acylated Serine Derivatives: A Unique Class of Arthropod Pheromones of the Australian Redback Spider, *Latrodectus hasselti*. Angew Chem Int Ed 49:2037–2040. https://doi.org/10.1002/anie.200906312
